# Supplementary material for: Efficient Kerr soliton comb generation in micro-resonator with interferometric back-coupling
Source: Nat Commun. 2022 Mar 11;13:1292. doi: 10.1038/s41467-022-28927-z (PMC8917225; doi:10.1038/s41467-022-28927-z)
Supplement: Supplementary file 1 — Supplementary Information [file 41467_2022_28927_MOESM1_ESM.pdf]

# SUPPLEMENTARY INFORMATION FOR: EFFICIENT KERR SOLITON COMB GENERATION IN MICRO-RESONATORS WITH INTERFEROMETRIC BACK-COUPLING

J.M. Chavez Boggio, D. Bodenmüller, S. Ahmed, S. Wabnitz, D. Modotto, and T. Hansson

## Supplementary note 1: Analysis of homogeneous solutions, detuning relations and resonance width

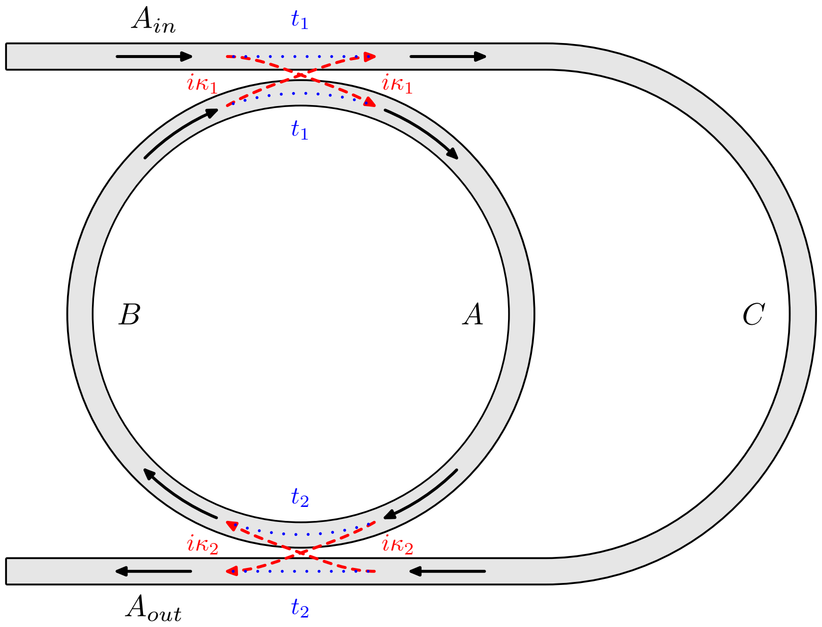


FIG. S1. Schematic of the microresonator device with optical feedback. The fields as well as the transmission and coupling coefficients are indicated at various locations.

The homogeneous solutions of the Ikeda map are found by considering the pump frequency only, and disregarding dispersive terms. The solution for propagation in each waveguide section is given by

$$A_m(L_1) = A_m(0)\exp\left[-\frac{\alpha_i L_1}{2} + i\gamma L_{eff}(L_1)|A_m(0)|^2\right] \tag{1}$$

where $L_{eff}(L_1) = (1 - e^{-\alpha_i L_1})/\alpha_i$, with analogous formulas for the fields $B_m(L_1)$ and $C_m(L_2)$. In the stationary case we must require the fields to reproduce themselves after each roundtrip. The roundtrip index can then be dropped, to obtain

$$A(0) = i\sqrt{\theta_1}A_{in} + \sqrt{1-\theta_1}e^{-i\delta_1}B(L_1), \qquad C(0) = \sqrt{1-\theta_1}A_{in} + i\sqrt{\theta_1}e^{-i\delta_1}B(L_1), \tag{2}$$

for the coupling conditions at node 1, and

$$B(0) = \sqrt{1-\theta_2}e^{-i\delta_1}A(L_1) + i\sqrt{\theta_2}e^{-i\delta_2}C(L_2) \tag{3}$$

for the coupling condition at node 2. By introducing the shorthand notation $t_j = \sqrt{1-\theta_j}$ and $\kappa_j = \sqrt{\theta_j}$, and the power dependent functions

$$a = t_1 - t_2 g_A g_B, \qquad c = \kappa_1 + \kappa_2 g_B g_C, \tag{4}$$

where $g_k = \exp[-(\alpha_i/2)L_j - i\delta_j + i\gamma L_{eff}(L_j)I_k]$ with $j = 1$ if $k = A, B$ and $j = 2$ if $k = C$. The coupling conditions can be rewritten as equations for the fields at $z = 0$ as

$$A(t_1 a + \kappa_1 c) = icA_{in}, \qquad C(t_1 a + \kappa_1 c) = aA_{in}, \tag{5}$$

$$g_B B(t_1 a + \kappa_1 c) = i(t_1 c - \kappa_1 a)A_{in}, \qquad A_{out}(t_1 a + \kappa_1 c) = (t_2 a g_C - \kappa_1 c g_A)A_{in}. \tag{6}$$

These equations can be used to solve for the powers, $I_A = |A|^2$, $I_B = |B|^2$, $I_C = |C|^2$, $I_{in} = |A_{in}|^2$ and $I_{out} = |A_{out}|^2$, so that one obtains the following closed system

$$I_A = \frac{|c|^2}{|t_1 a + \kappa_1 c|^2} I_{in}, \qquad I_C = \frac{|a|^2}{|t_1 a + \kappa_1 c|^2} I_{in}, \qquad I_B = \frac{|t_1 c - \kappa_1 a|^2}{|g_B|^2 |t_1 a + \kappa_1 c|^2} I_{in}. \tag{7}$$

These powers are related through the conditions $I_{in} + |g_B|^2 I_B = I_A + I_C$, $I_{out} + I_B = |g_A|^2 I_A + |g_C|^2 I_C$, and directly give the solution in the linear case (i.e., for $\gamma \to 0$).

In order to retrieve the detuning relations, we consider the phase shifts acquired by the field during propagation through section A and C, which are given by

$$\phi_1 = \beta L_1 \approx (\beta_0 + \beta_1 \Delta\omega) L_1 = 2\pi m_1 - \delta_1, \tag{8}$$

$$\phi_2 = \beta L_2 \approx (\beta_0 + \beta_1 \Delta\omega)(3L_1 + \Delta) = 2\pi m_2 - \delta_2, \tag{9}$$

where $\beta = \beta(\omega)$ is the propagation constant, $\beta_0 = \beta(\omega_0)$ and $\Delta$ is the tunable difference in length which is provided by the feedback arm. We have that $\beta_0 L_1 = 2\pi m_1$, $3\beta_0 L_1 = 2\pi m_2$, and $m_2 = 3m_1$. The detuning is related to the angular frequency difference $\Delta\omega$ between the pump and the resonance frequency $\omega_0$ through

$$\delta_1 = -\beta_1 L_1 \Delta\omega, \tag{10}$$

$$\delta_2 = -3\beta_1 L_1 \Delta\omega - \beta_0 \Delta - \beta_1(\Delta\omega)\Delta \cong 3\delta_1 - \beta_0 \Delta = 3\delta_1 + \delta_{20}, \tag{11}$$

where $\delta_{20} = -\beta_0 \Delta$ is a detuning offset. The propagation time in each section is given by $t_1 = \beta_1 L_1$ and $t_2 = \beta_1(3L_1 + \Delta) = 3t_1 + \beta_1 \Delta$. With the latter being related to the walk-off through $3L_1(\Delta\beta_1) = \beta_1 \Delta$.

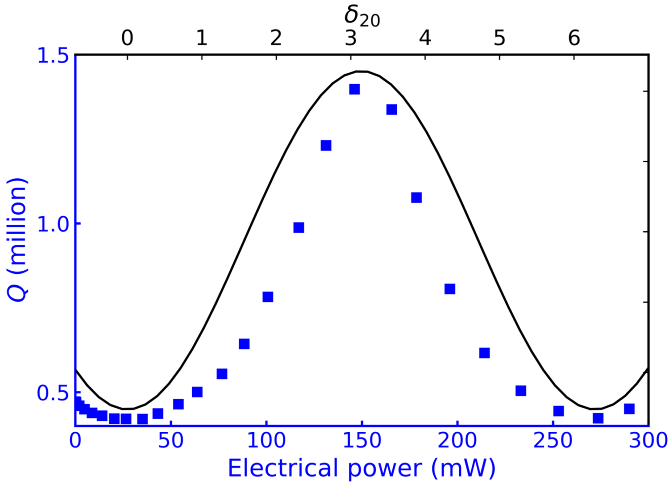


FIG. S2. Interferometric tuning of the coupling condition in resonator A. The measured average $Q$-factor as a function of the electrical power in the thermal heater is depicted by blue squares, while the calculated values from Equation (12) are shown by a solid black curve.

The resonance width can be determined from the expression for $I_B$ in Eq. (7). The resonance occurs when the imaginary part of the denominator is zero, while the width is obtained by setting the imaginary part equal to the real part. The resonance width is found to be approximately given by

$$\Delta\omega_T = [\alpha L_1 + (\theta_1 + \theta_2)/2 + \sqrt{\theta_1 \theta_2} \cos(\beta_0 \Delta)]/\beta_1 L_1, \tag{12}$$

where $\alpha_i L_1$ is the absorption loss contribution to the linewidth, while the second and third terms account for the contribution of the coupling coefficients. By adjusting $\Delta$, the coupling condition is tuned, which changes the linewidth of the resonance, and also changes the loaded $Q$-factor: $Q = \omega_0/\Delta\omega_T$ [1,2].

Figure S2 shows, for resonator A, the measured average loaded $Q$-factor variation as a function of the electrical power in the micro-heater (blue squares), as well as the calculated values (black line) using Equation (12). As can be seen, the agreement between measurements and theory is very good. The dependence of the average loaded $Q$-factor on $\Delta$ ($\delta_{20}$) follows a very similar behaviour to that of the other resonator with feedback (resonator B), which is shown in Figure 6(a) in the main text: this testifies of the high fabrication uniformity.

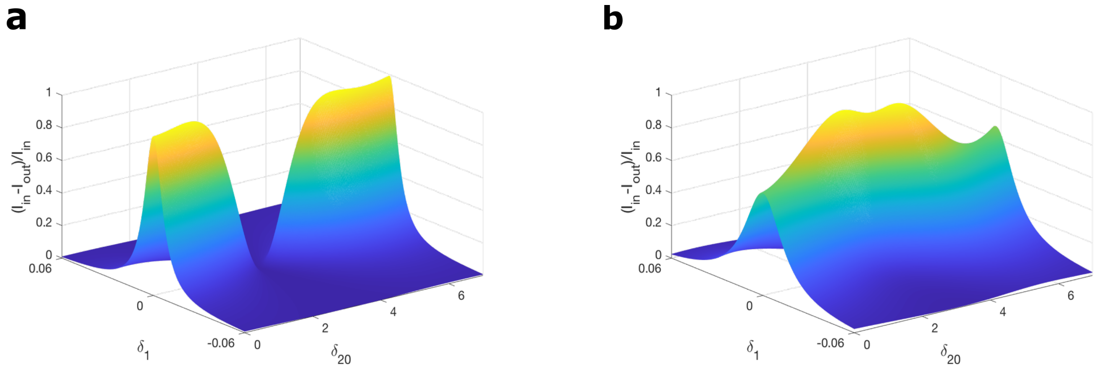


FIG. S3. Dependence of critical coupling on $\delta_1$ and $\delta_{20}$, when the coupling coefficients are (a) $\theta_1 = \theta_2 = 4.1 \times 10^{-3}$ or (b) $\theta_1 = 4.1 \times 10^{-3}$ and $\theta_2 = 2.38 \times 10^{-2}$. $I_{in}$ and $I_{out}$ are the input and output intensities, respectively. The calculation corresponds to the linear propagation regime.

In order to analyze the dependence of critical coupling on the detuning values $\delta_1$ and $\delta_{20}$, we consider in the linear regime two different coupling situations. Figure S3(a) shows the power coupled into the ring resonator as $\delta_1$ and $\delta_{20}$ are varied, and where $\theta_1 = \theta_2 = 4.1 \times 10^{-3}$, while Figure S3(b) shows the case when $\theta_1 = 4.1 \times 10^{-3}$ and $\theta_2 = 2.38 \times 10^{-2}$. When the coupling coefficients are identical, there is a clear dependence of the critical coupling on $\delta_{20}$: for $\delta_{20}$ close to $\pi$, pump power is not coupled into the ring but is reflected; whereas for $\delta_{20}$ close to 0, critical coupling is achieved. On the other hand, when the coupling coefficients are dissimilar, the contrast between critical coupling and the reflection regime is much less sharp: the resonator with feedback exhibits large amounts of coupling into the ring for nearly all values of $\delta_{20}$. This behavior helps to explain the results in Figure 6(a), where the conversion efficiency was observed to exhibit relatively little variation for the whole range of values of the cavity phase mismatch $\delta_{20}$.

**Supplementary note 2: Resonator with feedback characterization: transmission, coupling coefficients and chromatic dispersion**

In this supplementary note, we provide a detailed characterisation of the resonators with feedback that we used to generate DKSs. Two nominally identical chips were used: in chip 1 wire bonding was added, which allowed to apply an electrical current to thermal heaters, and permitted to tune the relative phase between the cavities. Whereas chip 2 had no wire bonding added. The experimental results in Figures 2, 3, 4, and 5(c-j) in the main text were generated with the chip without wire bonding. On the other hand, experimental results in Figures 5(a,b) and 6(a) in the main text were generated with the chip with wire bonding. To experimentally characterize the transmission properties of the resonators with feedback, a frequency-sweeping interferometric technique was employed [3]. This technique allows us to measure the resonance linewidth and depth, the FSR dispersion, and $Q$-factors as a function of wavelength. The light of a wavelength-tunable laser with 5 mW CW power is swept from 1550 to 1630 nm with a 10 nm/s speed. The light beam is split in two, 50 % of it is used to scan the resonances of the resonator with feedback, and the other 50% is injected into a Mach-Zehnder interferometer for generating a low frequency ruler with $\sim$ 20 MHz periodicity. Any irregularity of the laser scan velocity, when measuring the resonances, is tracked by the frequency ruler [4]. The transmitted spectrum through the resonator with feedback and the frequency ruler are both visualized with an oscilloscope at a frequency sampling rate of one point/MHz.

Figure S4(a) shows a zoom-in of the resonance pumped to generate the frequency comb spectra in Figures 2(e-h) and Figures 5(a,c,h) (i.e. resonator A). Note that the resonance does not exhibit a Lorentzian shape, but is distorted owing to the interferometric coupling. The signal obtained from the Mach-Zehnder interferometer having a $\sim$ 20 MHz periodicity is shown on the bottom. Figure S4(c) shows the zoom-in of the resonance pumped to generate the frequency comb power traces in Figures 4(a-d) and the frequency comb spectra in Figures 5(b, d-g, i-j) (i.e. resonator B). The resonances in Figures S4(a) and S4(c) are fitted in order to extract their widths and extinction ratios, and

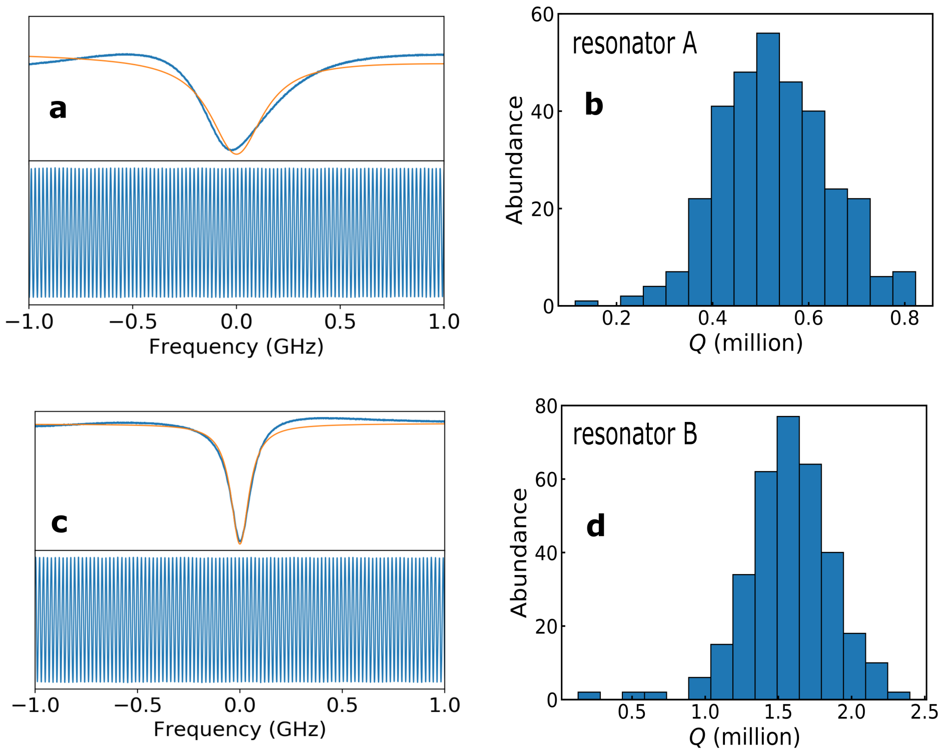


FIG. S4. Resonance characterization of chip 2. (a,b) resonator A and (c,d) resonator B. (a,c) Zoom of resonance at 1569.3 nm. (b,d) corresponding $Q$-factor abundances by scanning the resonances from 1550 to 1630 nm. This chip has no wire bonding added.

to retrieve the loaded $Q$-factor ($0.63 \times 10^6$ and $1.8 \times 10^6$, respectively). Figures S4(b) and S4(d) show the loaded $Q$-factor abundance from the 330 resonances scanned from 1530 to 1630 nm for the resonators A and B, respectively. The results in Figure S4 are obtained for chip 2.

### 2.1 Coupling coefficients

The two coupling coefficients of a resonator with feedback cannot be directly extracted from transmission scans, as it occurs in the case of an isolated resonator with a single coupling coefficient. Therefore, we retrieved the coupling coefficients by combining transmission scans and numerical simulations. In order to assess the correctness of this approach, we may first compare, for the case of isolated ring resonators (i.e., with just one coupling region), the experimentally measured coupling coefficients with the corresponding numerically calculated values.

The transmission coefficient of the coupler, $t$, is related to the loaded $Q$-factor and the round trip amplitude transmission, $a$, via

$$\frac{\sqrt{ta}}{1-ta} = \frac{Q\lambda_0}{L_1 \pi n_g} \tag{13}$$

where $L_1$ is the circumference of the resonator, $n_g$ is the silicon nitride material group index, and $\lambda_0$ the resonance wavelength. The round trip transmission, $a$, of the resonator is related to the intrinsic $Q$-factor, $Q_{int}$, by

$$\frac{\sqrt{a}}{1-a} = \frac{Q_{int}\lambda_0}{L_1 \pi n_g} \tag{14}$$

From the resonance extinction, $T$, and width, $\Delta\omega_T$, the intrinsic quality factor is obtained:

$$Q_{int} = \frac{2}{(1 \mp \sqrt{T})} \frac{\omega_0}{\Delta\omega_T} \tag{15}$$

In Equation (15), the plus sign is used in the case of an over-coupled resonance, while the minus sign is used in the case of under-coupled resonance. The coupling coefficient $\kappa$ is then retrieved from the transmission coefficient via $\kappa = \sqrt{1-t^2}$.

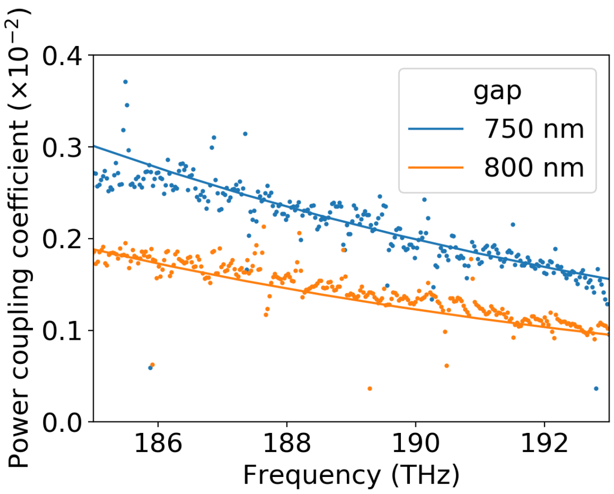


FIG. S5. Calculated (solid line) and experimentally (dots) obtained power coupling coefficient for two isolated resonators having gap of either 750 nm (blue) or 800 nm (red), respectively.

Numerical simulations were performed with the BeamProp package from RSoft, which implements a beam propagation method. We simulated only the coupling section consisting of a straight bus waveguide and the curved ring section. The nominal transverse dimensions and refractive index provided by the manufacturer of the chip are used as the waveguide parameters. The initial launch field distribution was chosen by ensuring that the total power, normalized to one, was located in the fundamental mode of the bus waveguide. Next, the power in the fundamental mode of the ring section at the end of the coupling section was extracted, in order to calculate the coupling coefficient.

Figure S5 shows the calculated (solid line) and experimentally (dots) obtained power coupling coefficient for two isolated resonators having $R = 800\,\mu$m, core width 1500 nm, core height 825 nm, and gap 750 nm (blue) or 800 nm (red), respectively. The agreement between experiments and simulations is very good, showing the correctness of the approach, as well as the accuracy of the nominal waveguide parameters of our chip (cross section, gap, and refractive index values).

After this experimental validation of our calculations, the coupling coefficients were calculated as a function of wavelength for the same parameters of the resonator with feedback that we used in the experiments, i.e., having $R = 800$ $\mu$m, core width 1500 nm, core height 825 nm, and gaps 650 nm (blue), 460 nm (green), and 410 nm (red), respectively. The results are shown in Figure S6, which are later used as the input parameters, in order to perform the Ikeda model simulations.

### 2.2 Dispersion

The chromatic dispersion of the resonator with feedback was retrieved from the transmission scans performed over a 1550 - 1630 nm wavelength range. The resonance frequencies, $\omega_\mu$, can be expressed through a Taylor expansion centered at the pumped mode $\omega_0$ as

$$\omega_\mu = \omega_0 + D_1\mu + \frac{1}{2}D_2\mu^2 + \frac{1}{6}D_3\mu^3 + ... \tag{16}$$

where $\mu$ is the relative mode index, $\frac{D_1}{2\pi}$ is the free-spectral range (FSR) of the microresonator, while $D_2$ and $D_3$, are related to the second- and third-order dispersion. The chromatic dispersion can be expressed through the frequency deviations from an equidistant grid centered at the pumped mode as

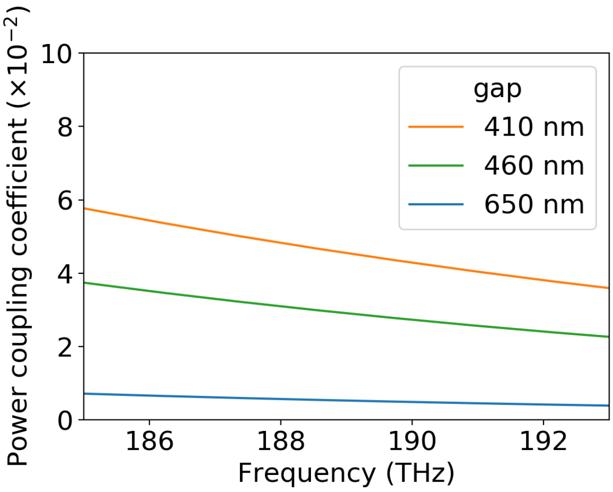


FIG. S6. Calculated coupling coefficient as a function of wavelength of the resonator with feedback for several gap values.

$$D_{int}(\mu) = \omega_\mu - (\omega_0 + D_1\mu) = \Sigma_{i>1} D_i \mu^i / i!, i \,\epsilon\, \mathbb{N}. \quad (17)$$

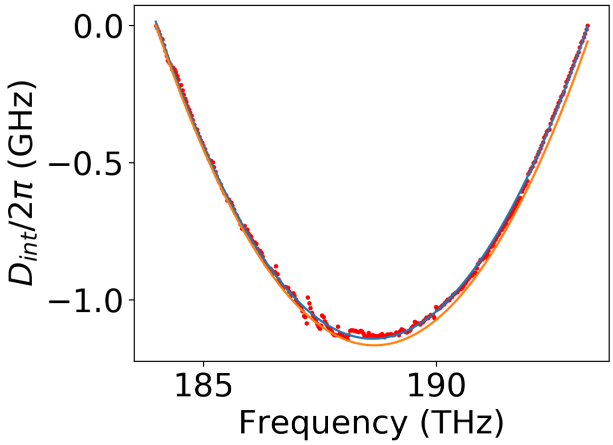


FIG. S7. The deviations from equidistant frequency grid of the measured resonances are shown by red dots. The fit of the resonances provides the experimentally measured $D_2$ coefficient (blue fitting line). The orange line shows the numerically calculated $D_{int}$ coefficient using the nominal parameters of our resonator: the agreement with the measurement is excellent, showing the good accuracy of the nominal parameters used along this investigation.

Red dots in Figure S7 show the measured dispersion of resonator B in chip 2 (no wire bonding added). The plot also contains the quadratic fit of the resonances, which leads to the experimental measured $D_2 = 0.538$ MHz (blue solid line), as well as the numerically calculated $D_2 = 0.534$ MHz, as obtained from the nominal manufacturer parameters (cross section and refractive index). By comparing with the measurement for the contiguous resonator A shown in Figure 2(b) in the main text, it can be noted that the chromatic dispersion is almost the same, indicating that good fabrication uniformity is achieved over the chip size. The $D_2$ coefficient relates to the waveguide dispersion (expressed by $\beta_2$ and $\beta_1$) of a ring resonator with a circumference of length $L$ via the equation $D_2 = -(2\pi/L)^2\beta_2/\beta_1^3$. Therefore, we may retrieve the experimental $\beta_2$ value for resonators A and B as -118.7 and -118.5 ps$^2$/km, respectively.

We have also measured the chromatic dispersion in a standalone microring resonator which is fabricated contiguously to a resonator with feedback in the same chip: the dispersion depends mainly on the transversal dimensions and is minimally impacted by the presence of the feedback section through the second coupling node.

## Supplementary note 3: Conversion efficiency vs number of propagating solitons

Very high conversion efficiencies were obtained for soliton crystals comprising around 50 pulses circulating in the ring of a microresonator with optical feedback. On the other hand, in isolated resonators it is not possible to reach high conversion efficiencies even when 50 pulses propagate in the resonator. Although we could not experimentally generate a small number of solitons, due to the thermo-optic effect in silicon nitride, we describe here by numerical simulations how the conversion efficiency varies with the number of circulating solitons. For the same parameters as in Figure 3 ($\delta_1 = 0.033$), we propagated a varying number of pulses in a resonator with feedback. After 10000 round trips, the pulses have evolved into DKSs, in all cases. Figures S8(a,b) and (c,d) show the cases for 10 and 1 solitons, respectively. The spectra at the output (top), ring (middle), and feedback (bottom) sections show the evolution of the frequency comb for both cases. While the conversion efficiency is reasonably good for the 10-soliton case, for the single-soliton case the efficiency is only below 1%.

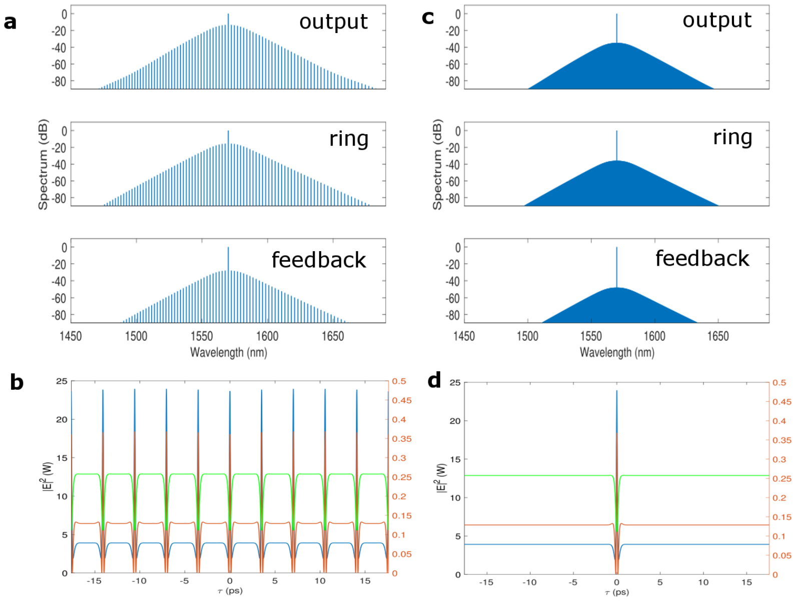


FIG. S8. Frequency comb spectra after propagation over 10000 round-trips of (a) ten equidistant pulses (c) one pulse. The spectra are calculated at the output (top), ring (middle), and feedback (bottom) sections. The corresponding temporal fields are shown in (b) and (d): output (red), ring (blue), and feedback (green) sections.

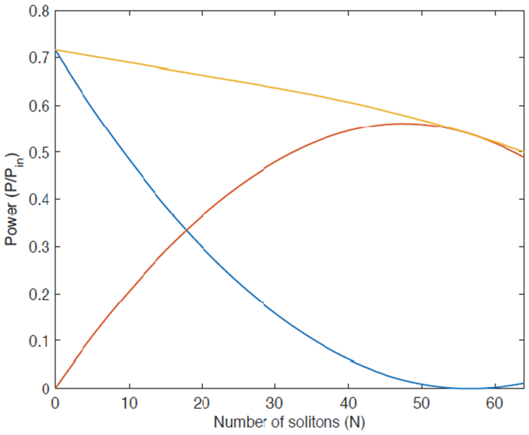


FIG. S9. Conversion efficiency as a function of the number of solitons. Calculated power carried by the pump (blue), comb lines (red) and entire comb (yellow).

Figure S9 shows the calculated conversion efficiency as a function of the number of solitons. The calculated power carried by the pump, comb lines, and entire comb is shown with blue, red, and yellow solid lines, respectively. The simulation was performed using parameters of the resonator with feedback as in Figure 3 in the main text and Figure S8, for the case with $\theta_2 = 3.7 \times 10^{-2}$. The results of Figure S9 show that the conversion efficiency is an approximately parabolic function of the number of solitons in the cavity. With the addition of each soliton, a small fraction of the power in the pump line is converted into power carried by the comb lines. The total output power is highest for the case of a pure homogeneous state, and decreases because of the losses needed to sustain the circulating soliton pulses. As a result of this decrease, the maximum of the comb line efficiency (for $N$ = 47-48, see red curve) is slightly shifted from the minimum of the pump line power ($N$ = 56-57, see blue curve).

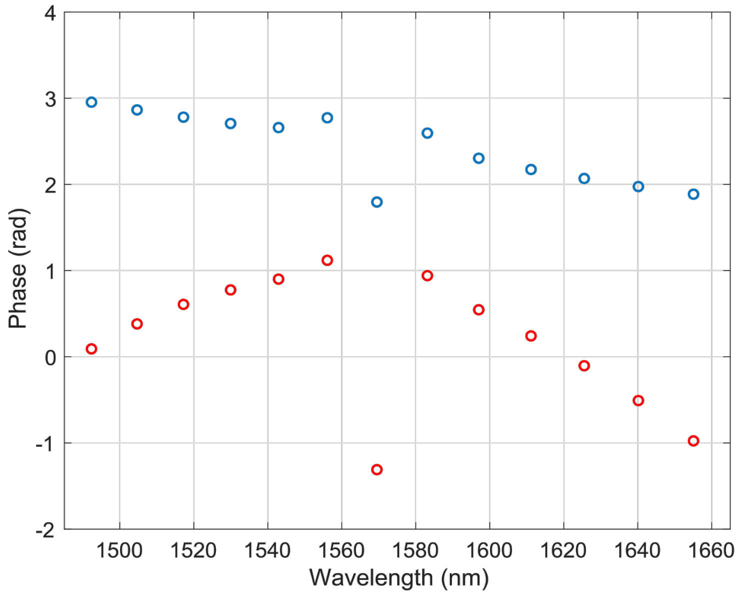


FIG. S10. Simulated phases, at the output of the resonator with feedback, of comb lines coming from either the microring (blue) or the feedback section (red).

To further demonstrate that indeed the observed high conversion efficiency comes from a destructive interference of the pump fields, we simulated the phases of all the comb lines from either the microring (blue) or the feedback section (red). These correspond to the same perfect soliton crystal that we show in the inset of Figure 3(h) of the main text. The results are plotted in Figure S10. As can be seen, there is a phase difference of $\pi$ for the pump lines, while the other comb lines have a smaller phase difference that varies with their wavelength.

### Supplementary note 4: Accessing single solitons

Multi-soliton steps were measured routinely in microresonators with optical feedback, as shown in Figure 4(a-d) of the main text. However, due to thermal effects, we could not access those states by direct tuning of the pump laser. Nevertheless, we could measure effects associated to the propagation of intense coherent pulses circulating in the ring: Raman self-frequency shift and third harmonic generation. Figure S11(a) shows the frequency comb spectrum obtained by pumping with 100 mW a resonator with feedback with the following parameters: gap1 = 650 nm, gap2 = 440 nm, FSR = 172 GHz, and loaded $Q$-factor of $10^6$. As can be seen, there is a small but noticeable Raman shift. The pump is only 17 dB stronger than the strongest comb line, indicating a moderate conversion efficiency. Figure S11(b) shows the frequency comb spectrum obtained by pumping with 400 mW a resonator with feedback having gap1 = 600 nm, gap2 = 440 nm, FSR = 172 GHz, and loaded $Q$-factor of $10^6$. The Raman shift can be clearly seen and it is around 1.4 THz, indicating the generation of intense pulses. Furthermore, the inset shows the picture of the chip taken during the frequency comb generation of the spectrum in Figure S11(b). It can be noted the scattering of strong green light, due to third harmonic generation in the ring resonator. This indicates that even though the frequency comb spectral shape in Figure S11(b) does not exhibit a perfect sech$^2$ profile, which is expected for pure DKS generation, a very intense and coherent pulse is circulating inside the ring, which gives rise to the observed Raman shift and third-harmonic green light.

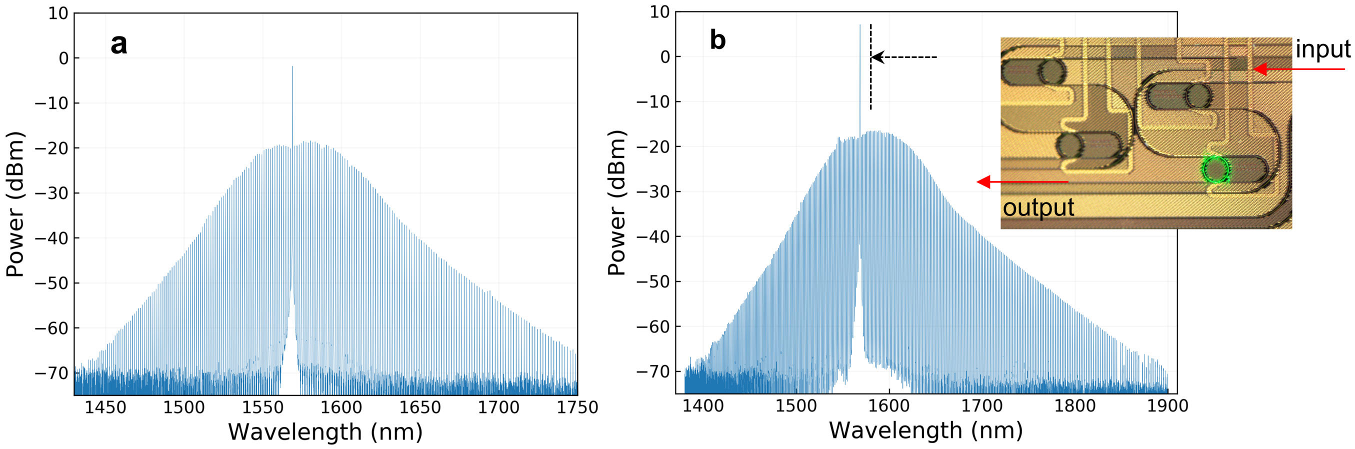


FIG. S11. Frequency comb spectra for: (a) 100 mW and (b) 400 mW. Inset: Picture of the chip showing green light generation due to a third-harmonic generation process originating from the intense pulses associated with the frequency comb in (b).

## Supplementary note 5: Comb states as a function of detuning

Figure S12 shows one of the traces of the generated comb power as a function of pump detuning from resonance as depicted in Figure 4(b), which was obtained with a pump power of 68 mW. The frequency comb spectra for three different detuning values are also shown, at points which are indicated with arrows. At low detuning, a Turing roll is generated; by increasing the value of the detuning, a modulation instability spectrum is obtained. By further increasing the pump detuning, a PSC is finally generated. The difference between the spectrum of a Turing roll and that of a PSC lies in the separation between the comb lines and the bandwidth, which is broader for the PSC.

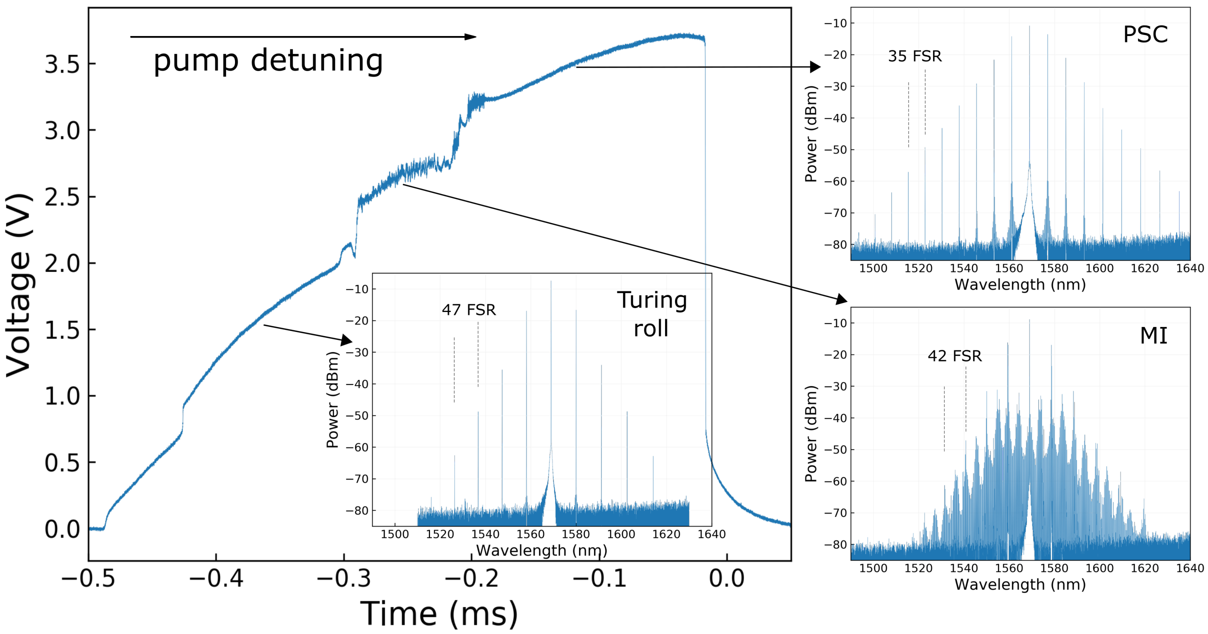


FIG. S12. Generated comb light as a function of pump-resonance detuning in a resonator with feedback from Figure 4(b) in the main text. The frequency comb spectra generated at different detuning values are indicated by an arrow.

## Supplementary note 6: Parametric threshold and PSC conversion efficiency vs pump power

From the various measurements performed with a resonator with feedback, a trend was observed: by keeping the phase mismatch between the main and the feedback cavities at a constant value, but varying the input pump power, the conversion efficiency could be changed. This observation is illustrated in Figures S13(b-e), where we plot the spectra of the generated perfect SCs for pump powers of 20, 30, 40, and 50 mW, respectively. These results come from the same set of measurements shown in Figure 4(a-d) in the main text. Note that, by reducing the pump power from 50 to 30 mW, the pump is progressively depleted due to the enhancement of the conversion efficiency. For a pump power of 20 mW this trend is slightly reversed. For a comparison, Figures S13(f) shows the frequency comb spectrum for a pump power of 40 mW, but for a pump detuning that generates a Turing roll: in this case the conversion efficiency is much smaller.

Interestingly, the threshold for parametric generation, i.e., for the appearance of the first comb lines, is at $\sim 15$ mW, as shown in Figure S13(a). This is slightly smaller than the expected value of 20.5 mW for a single resonator (i.e. for $f = 1$), and is at the edge of the experimental error in determining the pump power inside the chip.

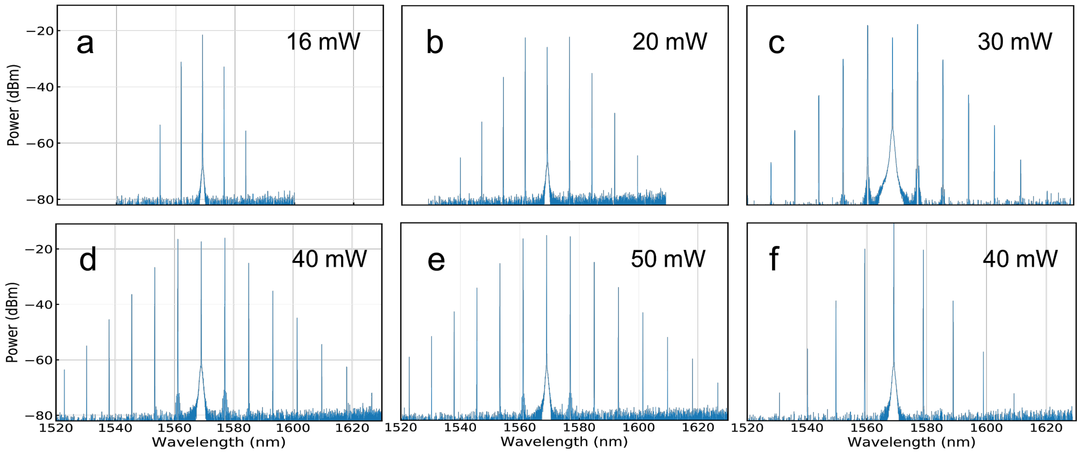


FIG. S13. (a-e) Generated frequency comb spectra for pump powers of 16, 20, 30, 40, and 50 mW, respectively. (f) shows the Turing roll generated for 40 mW of pump power.

## Supplementary note 7: Stability of the repetition rate

The coherence of frequency combs generated in our resonators with feedback was assessed through repetition rate measurements. The pump comb line was filtered out, and the whole comb spectrum was detected with a high-speed photodiode. The stability of the repetition rate was measured with a frequency counter, after performing a down-conversion from 28.4 GHz to sub-100 MHz. The frequency of the down-converted signal was measured with a frequency counter at different gate times. Every 5 minutes the gate time was changed. Figures S14(a-e) show the variation of the repetition rate over a time span of 30 minutes. A general trend that can be observed is a frequency drift towards smaller frequencies. Furthermore, jumps of the repetition rate frequency can be noted at $t = 755$ and at $t = 845$ seconds. Those jumps can be better observed for a gate time of 100 ms, when fast fluctuations are averaged out. Note that the fast fluctuations are a bit weaker at the beginning of the frequency comb operation. Interestingly, although the repetition rate signal of the frequency comb was always intense, the spectrum changed in a strong way. Figure S14(f) shows the spectrum one minute after the frequency comb is generated, while Figure S14(g) shows the OFC spectrum 15 minutes later. The strong lines have almost disappeared, indicating that a number of DKSs were annihilated. The comb spectrum in Figure S14(h) was taken ten minutes later: it exhibits a small change with respect to S14(g). Since our setup was not enclosed, it was subject to environmental disturbances, such as air flows, making it susceptible to drifts of the in-coupled pump power. Nevertheless, even though no stabilisation was performed, the robustness of soliton operation in the resonator with extended cavity turned out to be truly remarkable.

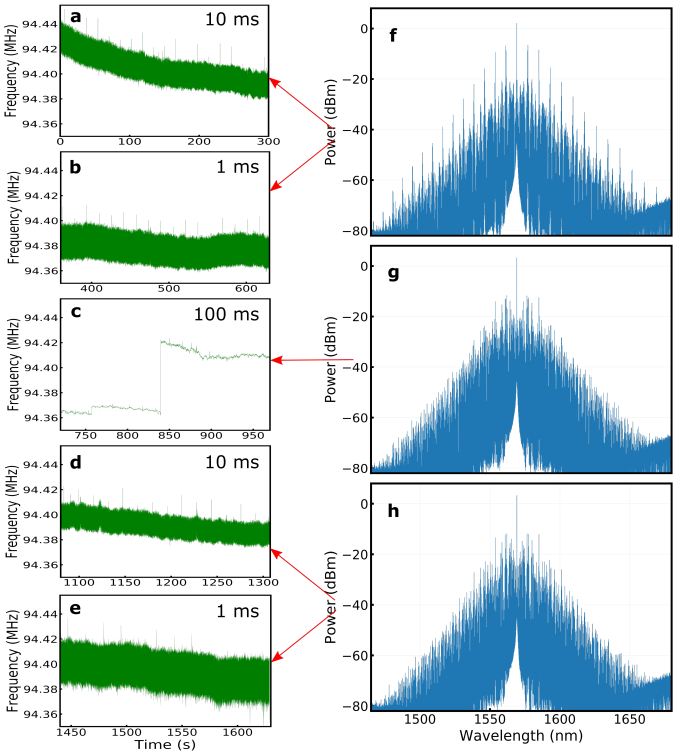


FIG. S14. (a-e) Repetition rate stability of the soliton crystal with defect measured with a frequency counter over 30 minutes. (f-h) Frequency comb spectral changes in time. Although high coherence was preserved, the spectrum changed, which might be due to soliton decays.

## Supplementary note 8: Inverse Fourier transform of experimental spectra

Frequency comb spectra reported in the main text were red-detuned and exhibit very intense repetition rate signals. Therefore, phase-locked comb lines and soliton formation are expected. Although we did not measure the phases of the comb lines or the auto correlation traces in order to characterize the generated temporal profiles, the reported spectra show features in the small comb lines that are very similar to those which were reported in the literature on soliton crystals. Because of this, we may assume as a working hypothesis that the strong comb lines are all in phase, while the weak ones are out of phase with respect to the strong ones, i.e. with a phase difference of $\pi$ [5,6]. Figures S15(a-d) show four frequency comb output spectra (left), along with their corresponding inverse Fourier transforms (right). The case of Figure S15(a) shows a perfect soliton crystal with moderate conversion efficiency, where its inverse Fourier transform shows 34 equidistant pulses. Note that there is no pedestal in the output pulses: this is related to the fact that the residual pump component has neither too large nor too small intensity, but exactly the appropriate one in order to cancel the background. For Figure S15(b) the inverse Fourier transform shows that a pulse is much weaker than all other 56 pulses, thus creating a vacancy. On the other hand, for Figures S15(c,d) the inverse Fourier transforms show that some pulses are weaker than all other pulses, so that the defects of these soliton crystals are more complex than the single vacancy case. Figure S15(e, top and middle panels) shows an Ikeda map simulation after 1000 round-trips, corresponding to the spectrum in Figure S15(b). For the input field we used the experimental spectrum, considering a flat phase, as already described for the weak and strong lines. It can be observed in the temporal profile at the ring and output sections, that there is a missing pulse, similar to the inverse Fourier transform calculation. Even though the simulated frequency comb spectrum matches well the experimental one (bottom panel), contrary to other soliton crystals with defects shown in Figure 5 in the main text, this crystal was unstable. The reason may be that the simulations did not include any avoided mode crossing, since we could not find conclusive experimental evidence for their location and strength. Such avoided mode crossing are often required in order to stabilize soliton crystals with defects.

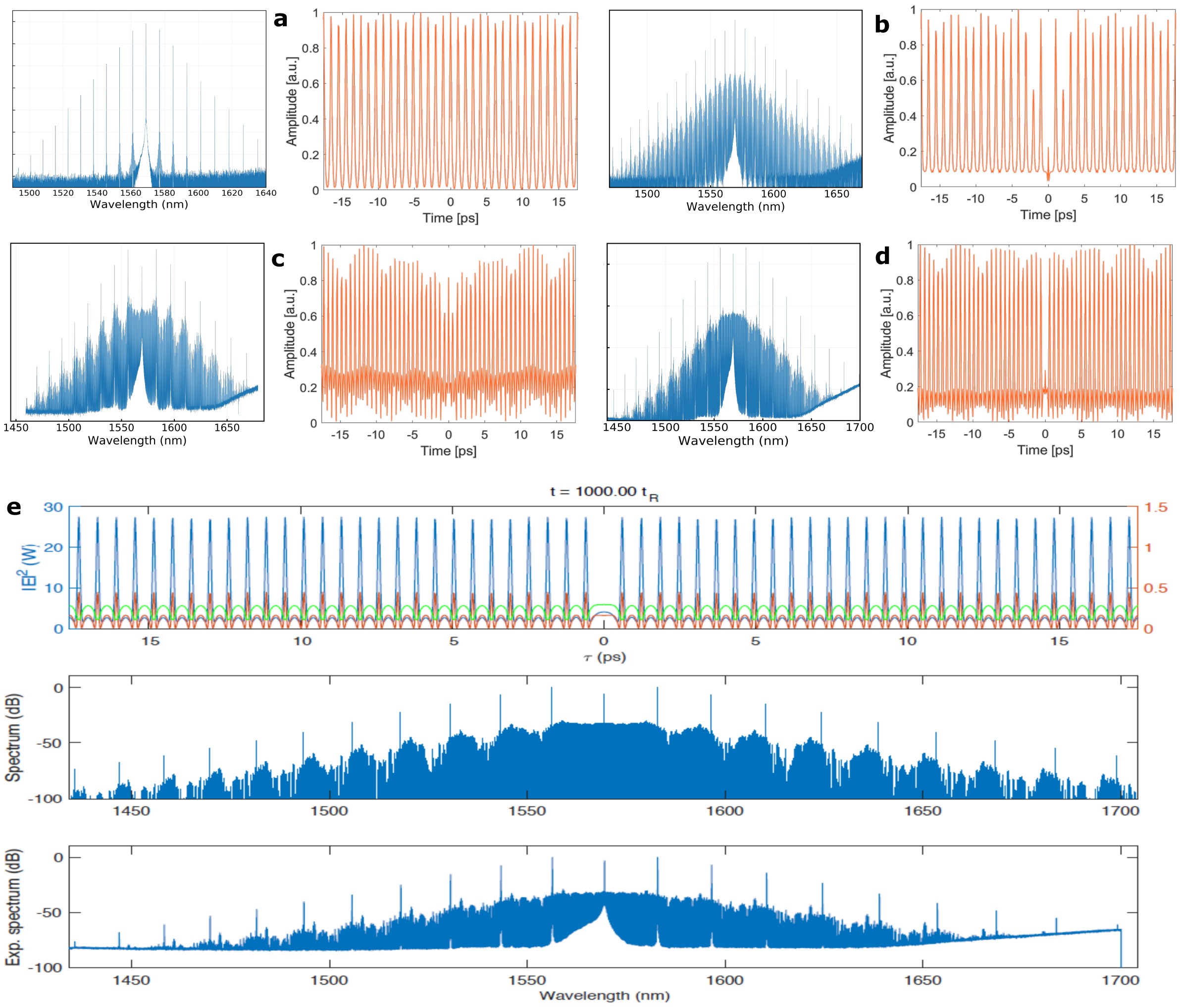


FIG. S15. (a-d) Frequency comb spectra (left) with their calculated inverse Fourier transform (right) whose period is 35.2 ps. (e) Ikeda map simulation (upper and middle panels) corresponding to the experimental frequency comb spectrum in the bottom panel

.

## Supplementary note 9: Conversion efficiency as a function of $\delta_{20}$

Perfect soliton crystal spectra obtained in resonator B by changing the value of $\delta_{20}$ with the thermal heater. The PSCs were generated by using a constant input pump power of 80 mW.

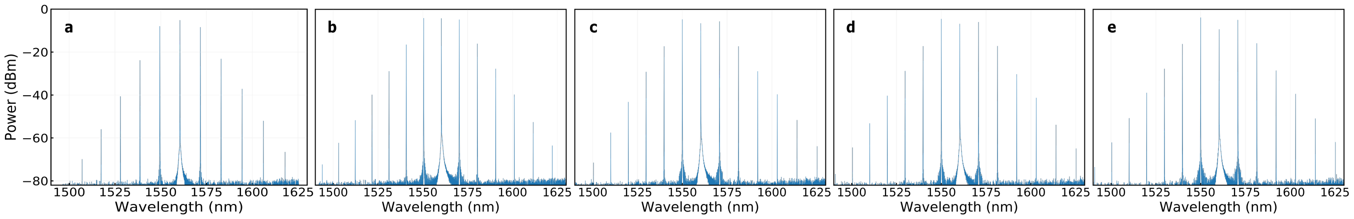


FIG. S16. Perfect soliton crystal spectra from which the conversion efficiency corresponding to Figure 6(a) in the main text was calculated

.

## References


[1] Yariv, A. Critical coupling and its control in optical waveguide-ring resonator systems. IEEE Photonics Technology Letters 14, 483-485 (2002).

[2] Chen, L., Sherwood-Droz, N., and Lipson, M. Compact bandwidth-tunable microring resonators. Opt. Lett. 32, 3361–3363 (2007).

[3] Pereira Cabral, A., Rebordão, J.M. Accuracy of frequency-sweeping interferometry for absolute distance metrology. Opt. Eng. 46 073602 (2007).

[4] Del'Haye, P., Arcizet, O., Gorodetsky, M. L., Holzwarth, R., and Kippenberg, T. J. Frequency comb assisted diode laser spectroscopy for measurement of microcavity dispersion. Nature Photon. 3, 529 (2009).

[5] Del'Haye, P., Coillet, A., Loh, W., Beha, K., Papp S. B., and Diddams, S. A. Phase steps and resonator detuning measurements in microresonator frequency combs. Nat. Commun. 6, 5668 (2015).

[6] Brasch, V. et al. Photonic chip based optical frequency comb using soliton Cherenkov radiation. Science 351, 357-360 (2015).
